# The Role of Natural Deep Eutectic Solvents in a Hydrogel Formulation Containing Lidocaine

**DOI:** 10.3390/pharmaceutics17030324

**Published:** 2025-03-02

**Authors:** Feria Hasanpour, Mária Budai-Szűcs, Anita Kovács, Rita Ambrus, Orsolya Jójárt-Laczkovich, Boglárka Szalai, Branimir Pavlić, Péter Simon, Levente Törteli, Szilvia Berkó

**Affiliations:** 1 Institute of Pharmaceutical Technology and Regulatory Affairs, Faculty of Pharmacy, University of Szeged, 6 Eötvös Str., H-6720 Szeged, Hungary; feria2010@yahoo.com (F.H.); budai-szucs.maria@szte.hu (M.B.-S.); gasparne.kovacs.anita@szte.hu (A.K.); ambrus.rita@szte.hu (R.A.); jojartne.laczkovich.orsolya@szte.hu (O.J.-L.); szalai.boglarka@szte.hu (B.S.); 2 Faculty of Technology, University of Novi Sad, Blvd. Cara Lazara 1, 21000 Novi Sad, Serbia; bpavlic@uns.ac.rs; 3 Institute of Pharmaceutical Chemistry, University of Szeged, Eötvös u. 6, H-6720 Szeged, Hungary; simon.peter.03@szte.hu (P.S.); torteli0515@gmail.com (L.T.)

**Keywords:** dermal permeation, hydrogel, lidocaine, local anesthetics, natural deep eutectic solvents (NADESs)

## Abstract

**Background/Objectives**: This study investigates the use of natural deep eutectic solvents (NADESs) in enhancing the solubility and skin permeation of a lidocaine base, a lipophilic form, in hydrogel systems. The aim was to develop an environmentally sustainable and biocompatible alternative to conventional lidocaine formulations, improving the dermal permeation and therapeutic efficacy. **Methods:** The lidocaine base was dissolved in a hydrophilic NADES system composed of choline chloride and citric acid, facilitating enhanced solubility, likely through new molecular interactions. Then, pH-adjusted hydrogels were formulated and optimized by employing a 3^2^ full factorial design. Raman and nuclear magnetic resonance (NMR) spectroscopy were applied to evaluate the stability of lidocaine in the optimal formulation. The biopharmaceutical properties were investigated using in vitro drug release and skin permeation studies. In vivo tests assessed physiological skin parameters such as the hydration and transepidermal water loss. **Results:** The developed NADES-containing hydrogel significantly improved the solubility and stability of lidocaine. Skin permeation studies demonstrated enhanced dermal permeation compared with conventional hydrogel and ointment. These improvements, namely the enhanced solubility of lidocaine in the formulation and its increased permeation, were attributed to the dual effect of the NADES. **Conclusions:** NADES-containing hydrogels represent a promising green technology for formulating lidocaine-containing dermal preparations. This approach offers a biocompatible, natural-based alternative that can enhance the bioavailability and efficacy of topical anesthetics.

## 1. Introduction

Lidocaine, an amide-based local anesthetic, has played a crucial role in pain management since its introduction in 1943 by Swedish chemist Nils Löfgren. Its rapid onset of action and moderate duration of effect make it particularly valuable in dermatological procedures, where effective pain relief is essential for patient comfort [1,2]. The primary mechanism of action involves the reversible blockade of voltage-gated sodium channels (VGSCs) in neuronal membranes by inhibiting the propagation of action potentials and effectively preventing the sensation of pain [3]. This blockade occurs when lidocaine binds to the inactive state of the sodium channels located in the dermis nerve endings, preventing sodium ions from entering the neuron and halting the depolarization process necessary for action potential generation [4,5].

The effectiveness of lidocaine is linked to its chemical structure, which allows it to exist in both ionized and non-ionized forms due to its amide group and basic nitrogen atom. This is crucial for its pharmacological action [6]. The non-ionized (non-protonated) form is lipophilic, enabling diffusion through the lipophilic stratum corneum, the outermost barrier of the skin cell membranes, whereas the ionized (protonated) form is more hydrophilic, which limits dermal absorption [7]. This balance between the two forms is influenced by the pH of the formulation, impacting the efficacy and duration of action of lidocaine. Additionally, the pH of the skin, typically ranging from 4.5 to 6.0, influences the ionization state of lidocaine, which has a pKa of approximately 7.9. At a pH of 4.5, a substantial portion of lidocaine exists in its ionized form, which, although water-soluble, has reduced permeability through the stratum corneum [8,9,10].

To enhance the penetration of lidocaine through the stratum corneum, various innovative strategies have been developed. Mechanical methods such as sonophoresis and microneedling significantly improve tissue penetration [11]. Similarly, iontophoresis, a needle-free technique, has demonstrated efficacy in pediatric surgical procedures [12]. Chemical enhancers, including surfactants, fatty acids, ethanol, and propylene glycol, disrupt the lipid bilayer of the stratum corneum, increasing the permeability for both hydrophilic and lipophilic drugs [13]. The transdermal delivery of lidocaine in microemulsion formulations has been improved by modifying the lipid organization within the stratum corneum [14]. Ethanol and linoleic acid also enhance the skin penetration of lidocaine by modifying the fluidity of the membrane [15]. A breakthrough in local anesthetics was the introduction of EMLA cream, a eutectic mixture containing lidocaine (2.5%) and prilocaine (2.5%), in the early 1980s. This formulation remains in a liquid state at room temperature, enhancing skin absorption. Studies have shown that EMLA cream significantly reduces pain during procedures compared with other anesthetic methods, such as lidocaine patches [16,17]. However, its onset of action typically requires 30–60 min under occlusive dressing, posing challenges in urgent situations [18].

In recent years, deep eutectic solvents (DESs) have received significant attention for their potential to serve as promising alternatives to conventional organic solvents and ionic liquids in a range of applications, including green chemistry, electrochemistry, and biotechnology [19,20,21]. DESs consist of a mixture of compounds (a hydrogen-bond acceptor (HBA) and a hydrogen-bond donor (HBD)) with a significantly lower melting point than each individual component. They can be classified into five types based on the chemistry of their components. Type I DESs are composed of a quaternary ammonium salt and metal chloride, offering a high solubilizing capacity for a wide range of compounds with potential toxicity and lack of biocompatibility, especially in biological systems. Type II DESs, made of a quaternary ammonium salt and metal chloride hydrate, share similar solubilizing properties to Type I but often exhibit lower viscosity compared to ionic liquids. Type III DESs, which consist of a quaternary ammonium salt and an organic hydrogen bond donor, such as amides or carboxylic acids, offer more benign toxicological profiles and higher biocompatibility. Type IV DESs, formed by metal chloride hydrates and hydrogen-bond donors, are highly customizable and are beneficial in extraction processes. Finally, Type V DESs are composed of natural materials that are entirely non-ionic, such as sugars and amino acids. This characteristic makes them particularly appealing for pharmaceutical applications. NADESs are a specific subgroup of DESs composed of naturally occurring components, such as sugars, organic acids, alcohols, amino acids, urea, choline chloride, and water [22,23,24].

Their “green” characteristics, biocompatibility, low cost, and stability have made NADESs a promising tool for drug development [19,25,26]. They have surged in various pharmaceutical applications as an innovative solvent system [27,28], including the extraction and dissolution of poorly soluble drugs. These solvents can be designed to improve the solubility of both hydrophilic and lipophilic compounds, providing a stable and less volatile alternative to traditional solvents. In previous studies, specific NADESs have been shown to improve the solubility of lidocaine, enabling effective transdermal drug delivery [29,30,31].

Computational chemistry techniques can be employed to model the interactions between lidocaine and the NADES components, providing insights into the solvation dynamics and stability of the resulting system. Molecular interactions are critical to the success of a formulation. Choline chloride, a widely used HBA, is a quaternary ammonium salt with favorable physicochemical properties, including high solubility in water, low toxicity, and biodegradability. It demonstrates excellent solubilizing properties due to its ability to form strong hydrogen bonds with HBDs like citric acid or lactic acid. This interaction leads to the formation of NADESs, which can effectively solubilize lidocaine by disrupting its crystalline structure and enhancing its molecular mobility. This innovative approach opens up new avenues for the development of effective topical anesthetic formulations [32,33,34,35].

For dermal application, hydrogels are often used as vehicles due to their favorable characteristics, such as their high water content, remarkable water retention capacity, and adjustable mechanical properties. Therefore, hydrogels can serve as an effective vehicle for NADESs. Hydrogels are user-friendly and provide a comfortable and soothing application experience. They are designed to absorb quickly into the skin without leaving a dry, greasy, or shiny residue, making them an ideal medium for drug delivery [36].

The objective of this study was to develop and optimize a novel formulation containing a hydrophilic NADES to dissolve a lidocaine base with low water solubility and incorporate it into the hydrophilic carrier system for dermal applications. The development and optimization of this system was based on a 3^2^ full factorial design. Although some studies have revealed the potential of various deep eutectic solvents to increase the solubility and stability of lidocaine, there is a lack of experimental studies that have investigated the effect of these natural solvents in pharmaceutical preparations, particularly in dermal drug delivery systems. A key focus was the characterization of this drug carrier system by X-ray diffraction (XRD) and Raman and nuclear magnetic resonance (NMR) spectroscopy. Additionally, we aimed to compare the developed system with traditional lidocaine formulations (hydrogel and ointment) in terms of their biopharmaceutical properties, including in vitro release and skin permeation studies. Furthermore, we investigated the effects of these formulations on the physiological parameters of the skin, such as the hydration and transepidermal water loss, through in vivo studies.

## 2. Materials and Methods

### 2.1. Materials

Natural deep eutectic solvent (NADES) mixtures were gifted from the Faculty of Technology, University of Novi Sad (Novi Sad, Serbia). Lidocaine, lidocaine hydrochloride, hydroxyethyl cellulose (HEC), sodium citrate (Na citrate), macrogol 400, and macrogol 1500 were obtained from Hungaropharma Ltd. (Budapest, Hungary). Acetonitrile (high-performance liquid chromatography (HPLC) grade) and H_3_PO_4_ (85%) (analytical grade) were purchased from VWR Int Ltd. (Radnor, PA, USA). Purified water (HPLC grade) produced with a TKA Smart2Pure system, TKA GmbH (Niederelbert, Germany) was used to prepare all the formulations. Purified and deionized water was used Milli-Q system, Millipore (Milford, MA, USA). Isopropyl alcohol was acquired from Molar Chemicals (Halásztelek, Hungary). Human skin was acquired from a Caucasian female patient who underwent an abdominal plastic surgery procedure at the Department of Dermatology and Allergology, University of Szeged. The investigations were performed with the approval of the Hungarian Medical Research Council (ETT-TUKEB, registration number: BMEÜ/2339-3/2022/EKU).

### 2.2. Methods

#### 2.2.1. Solubility Testing of Lidocaine in Natural Deep Eutectic Solvents

Seven NADES mixtures were selected as potential candidates for the solubilization of the lidocaine base. They were prepared by a simple heating and stirring method. Briefly, certain amounts of HBD and HBA were measured in a glass flask placed in a water bath at 80 °C on a hotplate and a magnetic stirrer. The mixtures were heated for several minutes (<10 min) until they reached the form of clear and transparent viscose liquid. The glass flask was further cooled to room temperature and the mixtures were preserved in liquid form [37].

Lidocaine was combined with the NADES mixtures at a ratio of 1:20 (lidocaine to NADES) and each mixture was stirred with a magnetic stirrer for approximately 24 h. Among the solvents tested, lidocaine demonstrated effective dissolution in several combinations, as presented in Table 1.

Among the tested solvents, sample S7 was selected for further investigations. This mixture (NADES (S7)) facilitated faster dissolution of lidocaine, achieving complete solubilization within 5 min. Additionally, it exhibited desirable properties, including the absence of a significant odor, a darker color, or a sticky consistency, making it a suitable candidate for further studies. This selection was guided by quality attributes, which are particularly important for dermally administered semi-solid formulations [38].

#### 2.2.2. Preparation of Lidocaine-Loaded NADES-Containing Hydrogel

The preparation of the hydrogel began with a design of experiments (DoE) approach to optimize the concentrations of NADESs and the pH-adjusting agent. This systematic investigation aimed to achieve a dermally acceptable pH range of 4 to 6 [39] and establish an appropriate viscosity for the formulations.

##### Design of Experiments (DoE)

The concentrations of the independent variables, namely the NADESs and the pH-adjusting agent (sodium citrate), were systematically evaluated using a 3^2^ full factorial experimental design, resulting in nine distinct formulations. The dependent variables, such as the pH and viscosity of the hydrogels, were analyzed, as outlined in Table 2.

##### Formulation Process for NADES-Containing Hydrogel

**Figure 1:**
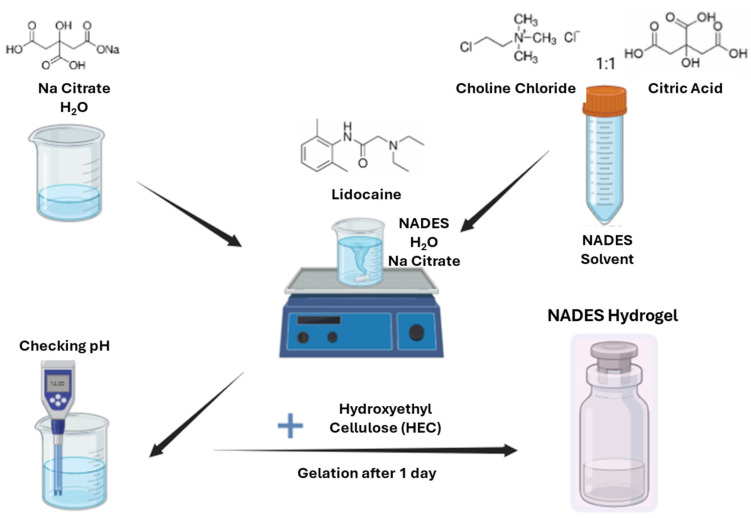
Illustration of the preparation process for lidocaine-loaded NADES-containing hydrogel. The image of the preparation was created with Biorender.com.

Different concentrations of NADES(S7), composed of choline chloride and citric acid in a 1:1 molar ratio, were used to dissolve 2 *w*/*w*% lidocaine. Additionally, sodium citrate (Na Citrate) was dissolved in purified water and incorporated into the formulations to adjust the pH, as suggested by the factorial design. Finally, the gels were prepared with 5 *w*/*w*% HEC [40]. After homogenization, the samples were stored in the refrigerator for one day to hydrate and homogenize uniformly. Then, the samples were considered ready for further investigations. Figure 1 visually represents the sequential steps involved in the formulation.

#### 2.2.3. Characterization of the NADES-Containing Hydrogel Formulations

##### Rheological Measurements

Rheological characterization of the formulations was performed on a Physica MCR302 rheometer (Anton Paar GmbH, Graz, Austria). The parallel plate geometry PP25 was utilized, with a measuring gap of 0.1 mm and a diameter of 25 mm. During the examination, the flow and viscosity curves were measured. The shear rate gradually increased from 0.1 to 100 1/s (up-curve) and subsequently decreased from 100 to 0.1 1/s (down-curve) in controlled rate mode. Each measurement lasted for 300 s and was performed at a temperature of 25 °C. The viscosity values were specifically obtained at a shear rate of 10 1/s. All the measurements were carried out in triplicate [41].

##### pH Measurements

A Testo 206 pH meter (Testo SE & Co. KGaA, Lenzkirch, Germany) with a pH2 probe was used to measure the pH value of 5 g of each sample. Three parallel measurements were carried out at room temperature [41].

##### X-Ray Diffractometry (XRD)

The crystalline structure of the selected NADES-containing hydrogel, along with its individual components (lidocaine, sodium citrate, and hydroxyethyl cellulose), was analyzed using XRD. The measurements were performed on a BRUKER D8 Advance diffractometer (Bruker AXS GmbH, Karlsruhe, Germany) equipped with Cu Kα radiation (λ = 1.5406 Å) and a VANTEC 1 detector. The scanning parameters were set to a voltage of 40 kV and a current of 40 mA. The angular range for the analysis was configured from 3° to 40° in 2θ, with a step size of 0.0073° and a step time of 0.1°/s. Data processing and analysis were carried out using EVA Software (version 13.0.0.1) (EVA Software Solutions, A223, Mumbai, India).

##### Raman Spectroscopy

The interaction between the components of NADES(S7) and lidocaine was analyzed by Raman spectroscopy. Samples were prepared and placed on aluminum-coated microscope slides, then investigated using a Thermo Fisher DXR Dispersive Raman Spectrometer (Thermo Fisher Scientific Inc., Waltham, MA, USA) equipped with a CCD camera. Raman spectra were collected for NADES(S7), lidocaine, and lidocaine dissolved in NADES(S7). The measurements utilized laser light at a wavelength of 780 nm, with an exposure time of 6 s and a total of 24 scans, incorporating corrections for cosmic rays and fluorescence. The laser power was set at 10 mW, and the slit width was maintained at 25 µm. Data acquisition and analysis were performed using the OMNIC™ 8.2 for Dispersive Raman software package (Thermo Fisher Scientific Inc., Waltham, MA, USA).

##### NMR Spectroscopy

To follow the chemical changes, the 1H NMR spectra were recorded in DMSO d6 solutions in 5 mm tubes at room temperature on a Bruker DRX-500 spectrometer with a 5 mm BBO Prodigy Probe (Bruker Biospin, Karlsruhe, Baden Württemberg, Germany) at 500 MHz (1H), with the deuterium signal of the solvent as the lock and tetramethylsilane (TMS) as the internal standard (1H). The samples (NADES(S7), NADES(S7) and lidocaine, NADES(S7) and lidocaine buffered with sodium citrate), to subdue the water signal, were lyophilized in advance.

#### 2.2.4. Preparation of Reference Formulations

Two reference formulations were selected to compare their efficacy in terms of the in vitro biopharmaceutical properties and in vivo physiological features of the skin.

- Reference hydrogel (Hydrogel Ref): It was prepared using the salt form of lidocaine (2 *w*/*w*% lidocaine hydrochloride) dissolved in water. A 5 *w*/*w*% concentration of HEC was incorporated to form the hydrogel matrix. After homogenization and uniform distribution of HEC, it was refrigerated for one day to facilitate complete gelation and homogeneous gel formation [42].
- Reference ointment (Ointment Ref): The ointment was prepared using a formulation that contained 400 and 1500 macrogol (polyethylene glycol) in a 1:1 ratio, along with 2 *w*/*w*% lidocaine as a reference formulation. First, the ointment base was melted at a temperature of 60–70 °C. Once the base was fully melted, lidocaine was added to the mixture and then the components were stirred continuously until the mixture cooled to ensure that the lidocaine was evenly dissolved in the ointment base [43].

#### 2.2.5. In Vitro Release and Skin Permeation Tests

The in vitro drug release (IVRT) and in vitro drug permeation (IVPT) were assessed using a Franz diffusion cell (Phoenix RDS automatic diffusion system, Teledyne LABS, Thousand Oaks, CA, USA) consisting of a donor and an acceptor compartment separated by either a synthetic cellulose acetate membrane or a heat-separated human epidermis (HSE). The cellulose acetate membrane (Porafil membrane filter, pore diameter: 0.45 μm, Macherey-Nagel GmbH & Co. KG, Düren, Germany) was pre-soaked in freshly prepared phosphate buffer (PBS, pH 7.4). The HSE was prepared by placing it in a water bath at 60 ± 0.5 °C to separate the epidermis from the dermis. Approximately 0.3 g of the semisolid formulation was applied to the membrane. The acceptor chamber contained 10 mL of PBS maintained at 32.5 °C at a stirring speed of 400 rpm. For the IVRT, the experiment was conducted for 6 h in six parallel cells with sampling times set at 0.5, 1, 2, 4, 5, and 6 h. For the IVPT, the duration was extended to 24 h with sampling times set at 0.5, 1, 2, 3, 4, 5, 6, 8, 10, 12, 16, 20, and 24 h.

The cumulative amount of lidocaine in the acceptor phase was analyzed using HPLC (Shimadzu Nexera X2 UHPLC, Kyoto, Japan) with a C18 reverse-phase column (ZORBAX Eclipse XDB-C18, Phenomenex, Torrance, CA, USA) with 5 µm and 4.6 × 150 mm dimensions. The mobile phase was 0.1% phosphoric acid (solvent A):acetonitrile (solvent B) 90:10 in gradient mode. It was changed from 90:10 (A: B, *v*/*v*) to 40:60 (A:B, *v*/*v*) in 6 min, and then back to 90:10 (*v*/*v*) between 6.1 and 10 min. The flow rate of 0.8 mL/min was set over 10 min, the column temperature and sample tray temperature were set at 25 °C, and the detection was made at 230 nm. The injection volume was 5 µL. The time of analysis was 10 min, and the retention time was 4.2 min.

According to the EMA guidelines [44], IVRT serves as an effective method for evaluating the release rate and extent of the active ingredient in formulations. The key parameters determined included the (a) drug release rate, calculated as the slope of the cumulative amount of the active substance released versus the square root of time during the linear portion of the drug release profile; and (b) cumulative amount released expressed in mass per surface area at the final sampling time within this linear range.

#### 2.2.6. In Vivo Hydration and Transepidermal Water Loss Tests

The Corneometer^®^ CM 825 (Courage and Khazaka Electronic GmbH, Cologne, Germany) probe measures skin hydration by employing capacitance technology, focusing on the outermost layer of the skin (stratum corneum) at a depth of 10–20 µm. This ensures highly accurate and reproducible results within one second, with minimal interference from surface residues [45]. The Tewameter^®^ TM 300 (Courage and Khazaka Electronic GmbH, Cologne, Germany) probe measures the transepidermal water loss (TEWL), a critical parameter for evaluating the skin’s barrier function. The probe indirectly measures the density gradient of water evaporation from the skin. Together, these probes enable a comprehensive analysis of the skin hydration and barrier function, making the system suitable for dermatological research and product testing [46,47].

During the measurements, changes in the skin condition of the forearms of six volunteers were examined. The investigations were performed with the approval of the National Public Health and Pharmaceutical Center (NNGYK, registration number: OGYÉI/56435-2/2023). The female volunteers were between 25 and 60 years old and had healthy skin. They had not used any cosmetics on their forearms in the 24 h prior to the measurement to avoid influencing the results. The untreated skin condition was measured at baseline. Then, 200 mg of the preparations was applied to the marked area, left for 30 min, the excess was removed, then corneometric and tewametric measurements were performed at 30, 60, 90, 120 and 150 min. The values of the untreated skin were taken as zero and the changes were expressed as a percentage compared to this value at each interval.

#### 2.2.7. Statistical Analysis

Statistical data analysis was performed using Prism for Windows software 5.0 (GraphPad Software Inc., La Jolla, CA, USA). The statistical difference between samples was analyzed using two-way ANOVA followed by the Bonferroni post-test.

Levels of *p* ≤ 0.05 *, *p* ≤ 0.01 ** and *p* ≤ 0.001 *** were significant versus the control. Six parallel measurements were conducted for the IVRT, IVPT, hydration, and transepidermal water loss tests.

## 3. Results

### 3.1. Results of the Design of Experiments (DoE)

Based on the design of experiments (DoE), Table 3 shows the values of the independent variables and the measured average values ± SDs of the dependent variables for all the samples.

#### 3.1.1. Evaluation of Rheological Measurements

**Figure 2:**
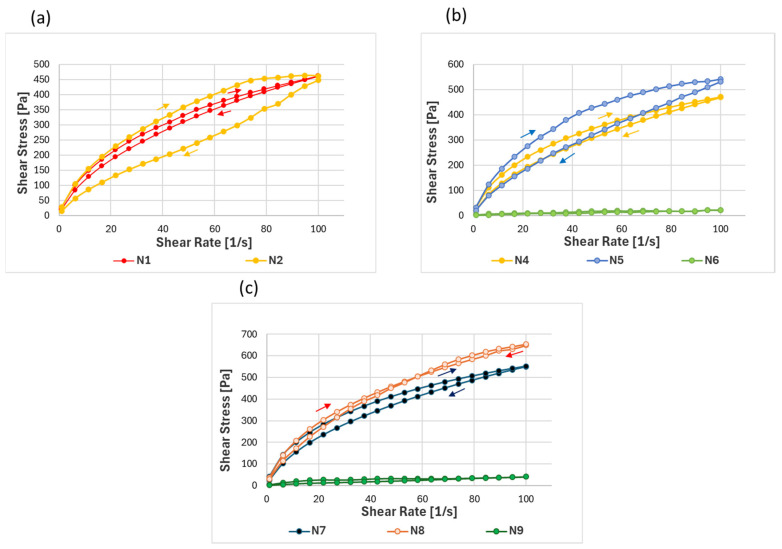
Flow curves of the hydrogel formulations of the DoE with varying concentrations of NADES and sodium citrate: (**a**) fixed 10 *w*/*w*% NADES with 8 and 13 *w*/*w*% sodium citrate; (**b**) constant 15 *w*/*w*% NADES with 8, 13, and 18 *w*/*w*% sodium citrate; and (**c**) fixed 20 *w*/*w*% NADES with 8, 13, and 18 *w*/*w*% sodium citrate.

Based on the DoE, the flow curves (Figure 2) of nine formulations were evaluated, with the viscosity values (Table 3) measured at a shear rate of 10 1/s utilized for the statistical analysis of the factorial design. The flow curves predominantly exhibited shear-thinning behavior, which facilitates smooth application under shear stress. In Figure 2a, the flow curves for formulations containing 10 *w*/*w*% NADES with sodium citrate concentrations of 8% (N1) and 13% (N2) demonstrated the effect of sodium citrate on the flow behavior of the hydrogel. Sodium citrate ionically interacted with the hydroxyl groups of hydroxyethyl cellulose, strengthening the polymer network and increasing the viscosity with an increasing concentration. Notably, N2 (13 *w*/*w*% sodium citrate) exhibited higher viscosity compared to N1 (8 *w*/*w*% sodium citrate) and displayed thixotropic behavior as evidenced by its hysteresis loop, which indicated a time-dependent recovery of viscosity once the shear rate was reduced. In contrast, N1 showed no hysteresis, indicating the immediate recovery of the structure under the shear. However, at the highest sodium citrate concentration of 18 *w*/*w*% (N3), precipitation occurred, which destabilized the hydrogel structure, and its flow curve was unmeasurable.

Similar trends were observed in Figure 2b,c, where increasing the NADES concentration to 15 *w*/*w*% (Figure 2b) and 20 *w*/*w*% (Figure 2c) further improved the viscosity of the formulations. This suggested that higher NADES content reinforced the hydrogel structure, making it thicker and more stable overall [48]. At the highest sodium citrate concentrations, formulations N6 and N9 also showed a significant decrease in viscosity. This indicated that excessively high concentrations of sodium citrate could destabilize the hydrogel matrix, likely due to the excessive ionic strength interfering with the polymer interactions. This phenomenon may have occurred because the ionic nature of sodium citrate influenced the electrostatic interactions with the polymer. The increased ionic strength may have neutralized the charges and reduced the repulsive forces between polymer chains, leading to more fluid-like behavior [49].

Overall, the lower sodium citrate concentrations in the NADES formulations demonstrated better spreadability while maintaining shear thinning behavior, supporting the finding that an ideal formulation should have the ability to return to its original apparent viscosity within a defined period, ensuring sustained physical stability over time [48]. A viscosity range of 5000–20,000 mPas is considered acceptable for dermal applications, as the stability and spreadability of hydrogels are strongly influenced by their viscosity [38]. The viscosity curves ae included in the Supplementary Material (Figure S1) to complement the shear stress data and support the values shown in Table 3. These curves provide a more detailed representation of the flow behavior, allowing for better interpretation of the rheological properties of the analyzed samples.

#### 3.1.2. Results of pH Measurements

This experimental study elucidated the relationship between the concentrations of NADES and sodium citrate (independent variables) and the pH of the formulations (dependent variable) within the framework of the DoE. The pH values of the formulations ranged from 3.63 to 5.05. The results indicated that an increase in the concentration of NADES led to a decrease in the pH, while elevating the concentration of sodium citrate resulted in an increase in the pH. This observation highlights the neutralizing effect of sodium citrate on the acidic environment induced by the NADES. The data supporting these findings are presented in Table 3.

#### 3.1.3. Statistical Analysis of the DoE

To optimize the formulation, a nine-run 3^2^ factorial design was used [50,51] to evaluate two factors: NADES concentration (X_1_) and sodium citrate concentration (X_2_), each tested at three levels (−1, 0, and +1). The measured responses were the pH (Y_1_) and viscosity (Y_2_). Statistical analysis was conducted using Statistica for Windows (version 13.5, Stat Soft Inc., Tulsa, OK, USA) with a 95% confidence interval and an alpha of 0.05. Table 3 summarizes the relationship between the independent variables (NADES and sodium citrate concentrations) and the responses (pH and viscosity).

Different models (linear, two-factor interaction (2FI), and quadratic) were tested to fit the response surface. The quadratic model was found to be effective in capturing nonlinear effects and interactions between the NADES and citrate salt concentrations.

To enhance the model accuracy, factors with minimal impact, identified by lower coefficients, were removed to maximize the adjusted R^2^ values. This step allowed for a more predictive model that accurately represented both the linear and quadratic effects on the responses. The final polynomial model is expressed in Equation (1).

(1) $$Y=\beta_{0}+\beta_{1}X_{1}+\beta_{11}{X_{1}}^{2}+\beta_{2}X_{2}+\beta_{22}{X_{2}}^{2}+\beta_{12}X_{1}X_{2}+\beta_{112}{X_{1}}^{2}X_{2}+\beta_{122}X_{1}{X_{2}}^{2}+\beta_{1122}{X_{1}}^{2}{X_{2}}^{2}$$

The response variables Y_1_ and Y_2_, representing the pH and viscosity of the formulations, respectively, are influenced by the independent variables X_1_ (NADES concentration) and X_2_ (sodium citrate concentration). In this model, β_0_ represents the intercept, which is the baseline response when all the independent variables are at their mean values. Coefficients β_1_ and β_2_ are the linear coefficients for X_1_ and X_2_, respectively, describing the primary effects of these factors on Y. Positive coefficients indicate synergistic effects, while negative coefficients reflect antagonistic effects.

The quadratic coefficients β_11_ and β_22_ account for the nonlinear effects of X_1_^2^ and X_2_^2^ on the response variable.

The interaction between X_1_ and X_2_ is captured by the interaction coefficient β_12_, which reflects how the combined influence of these two factors affects the response (Y). Higher-order interactions are represented by β_112_ and β_122_, which are the coefficients associated with X_1_^2^X_2_ and X_1_X_2_^2^, respectively. Finally, the coefficient β_1122_ corresponds to the term X_1_^2^X_2_^2^, which captures the combined influence of both factors at their higher levels. Equation (2) and Equation (3) represent these responses, the pH and viscosity, respectively.

**Figure 3:**
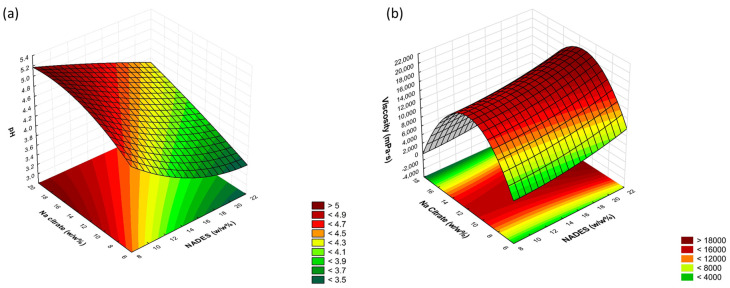
Effect of the sodium citrate and NADES concentrations on the pH (**a**) and viscosity (**b**) of the formulations.

The best equation for the pH was obtained with a two-way interaction and ignoring the interaction of X_1_^2^X_2_^2^ (quadratic NADES and sodium citrate concentration). The high R-squared value (0.9992) confirms that the NADES and sodium citrate concentrations are the primary factors in controlling the pH stability in this hydrogel. The adjusted R-squared value of 0.99356 confirms the high accuracy of the model in explaining the pH variation. The mean square residual (MS Residual) of 0.0013444 indicates a minimal average deviation between the observed and predicted pH values, confirming the precision of the model (Figure 3a).

(2) $$\begin{array}{cc} & pH\left(Y_{1}\right)=4.367778-0.396667X_{1}-0.028333{X_{1}}^{2}+0.341667X_{2}+0.034167{X_{2}}^{2}\\ & +0.030000X_{1}X_{2}-0.017500X_{1}{X_{2}}^{2}+0.025000{X_{1}}^{2}X_{2} \end{array}$$

The optimal equation for predicting viscosity was determined after ignoring the interaction term X_1_^2^X_2_. The significant factors included X_2_ (linear sodium citrate concentration with an inverse relationship) and X_2_^2^ (quadratic sodium citrate concentration with a positive relation) with an R-squared value of 0.99941 and an adjusted R-squared of 0.99528, which indicate both linear and quadratic effects. These factors, X_2_ and X_2_^2^, play an essential role in capturing the curvature of the response surface and accurately explaining the variability in viscosity (Figure 3b).

(3) $$\begin{array}{ll} Viscosity\left(Y_{2}\right)= & 9646.54+1420.363X_{1}-223.795{X_{1}}^{2}-5564.023X_{2}\\ & +4770.845{X_{2}}^{2}-264.955X_{1}X_{2}+685.978X_{1}{X_{2}}^{2}+277.721{X_{1}}^{2}{X_{2}}^{2} \end{array}$$

Based on these results, the N1 formulation with an NADES concentration of 10 *w*/*w*% and a sodium citrate concentration of 8 *w*/*w*% was chosen for further study. The formulation demonstrated a pH value of 4.46, which closely corresponded to the physiological pH range of human skin (4.5 to 6.0), thereby ensuring optimal biocompatibility and safety for dermal pharmaceutical applications [10,52]. The viscosity value of the N1 formulation was 11,000 mPa·s, which is suitable for application on the skin; additionally, lower viscosity has been reported to correlate with faster drug release [10,38,47].

### 3.2. Characterization of the NADES-Containing System

#### 3.2.1. Results of XRD Analysis

**Figure 4:**
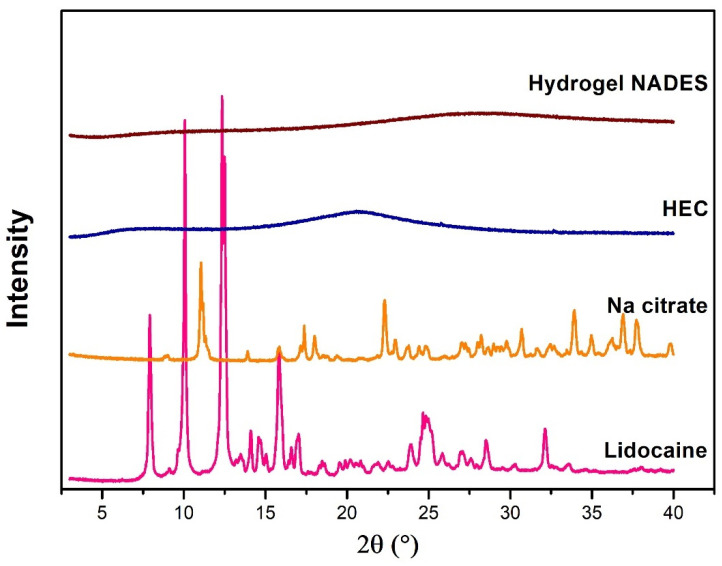
XRD diffractogram showing the crystalline profiles of raw lidocaine, sodium citrate (Na Citrate), hydroxyethyl cellulose (HEC), and Hydrogel NADES.

The N1 composition of the NADES-containing hydrogel (Hydrogel NADES) was prepared, and the diffraction patterns of the analyzed materials (hydrogel and raw materials) were evaluated using X-ray diffraction analysis. The lidocaine and sodium citrate exhibited sharp, well-defined crystalline structures. The diffraction pattern of lidocaine demonstrated several sharp peaks (at 7.69°, 10.12°, 12.7°, 15.95°, and 25.12° 2θ that were comparable with the Cambridge Structural Database (CSD version 2022.3.0). The reflections of sodium citrate at 11.1°, 17.5°, 22.5°, 29°, and 34° 2θ also indicated the presence of a crystalline pattern. However, the diffractograms of the Hydrogel NADES showed no distinct crystalline characteristics (Figure 4). This result suggested that the lidocaine was uniformly and molecularly dispersed within the hydrogel containing the NADES matrix, which is critical for achieving the desired biopharmaceutical profiles [53].

The hydrogels containing HEC displayed peaks in the range of approximately 2θ = 25–30° in their XRD patterns compared to the original peak, which appeared at 2θ = 21.47°. These peaks, associated with HEC, were not sharp, indicating the more amorphous nature of the cellulose backbone in the hydrogel matrix. The presence of broad peaks suggests that the crystallinity of the HEC component may be reduced upon incorporation into the hydrogel, which can enhance the flexibility and swelling behavior of the material [54,55,56].

#### 3.2.2. Results of Raman Measurements

**Figure 5:**
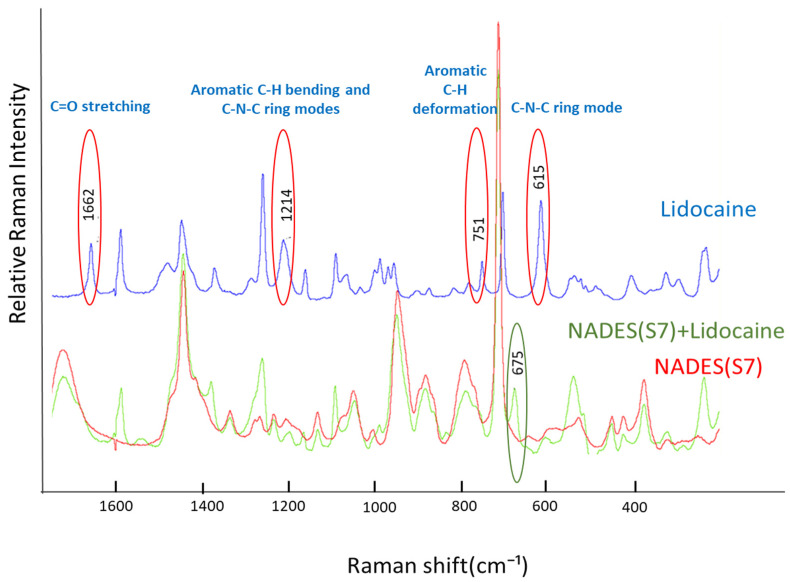
Raman spectra of NADES(S7) compared with the spectra of NADES(S7) + lidocaine and pure lidocaine.

The Raman spectra were analyzed to assess the interactions and the chemical stability of lidocaine in the NADES(S7) solvent (Figure 5). The disappearance of four Raman peaks at 1662 cm^−1^, 1214 cm^−1^, 751 cm^−1^, and 615 cm^−1^, along with the appearance of a new peak at 675 cm^−1^, after dissolving lidocaine in the NADES(S7) system indicated changes in the molecular structure of lidocaine. Specifically, the disappearance of the peak at 1662 cm^−1^, corresponding to the C=O stretching, the peak at 1214 cm^−1^ associated aromatic C–H bending and C–N–C ring modes, the peak at 751 cm^−1^ corresponding to aromatic C–H deformation, and the peak at 615 cm^−1^ associated with C–N–C ring mode suggested a modification or hydrolysis of the amide bond, potentially caused by interactions with the solvent [57].

According to the literature, the appearance of a new peak at 675 cm^−1^ is attributed to a shift in the peak at 706 cm^−1^ to a lower frequency in the Raman spectrum. This phenomenon may be linked to 2,6-dimethylaniline, a hydrolysis product of the amide bond in lidocaine, which is considered a toxic impurity and a carcinogenic metabolite [58,59]. However, this peak may also suggest the formation of a new bond interaction between lidocaine and the NADES(S7). Therefore, further investigation is required to determine whether the observed changes indicate chemical degradation or novel molecular interactions. To clarify this, we examined the samples using NMR spectroscopy. The NMR analysis was expected to provide further insight into whether degradation products, such as 2,6-dimethylaniline, were indeed formed or whether new bonding interactions were responsible for the observed Raman shifts [60].

#### 3.2.3. Results of NMR Measurements

**Figure 6:**
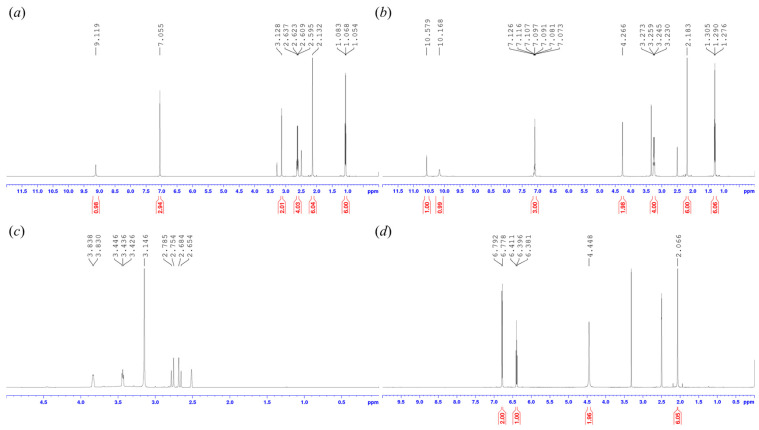
^1^H NMR spectrum of (**a**) lidocaine base, (**b**) lidocaine hydrochloride, (**c**) NADES(S7), and (**d**) 2,6-dimethylaniline in DMSO-*d*_6_.

**Figure 7:**
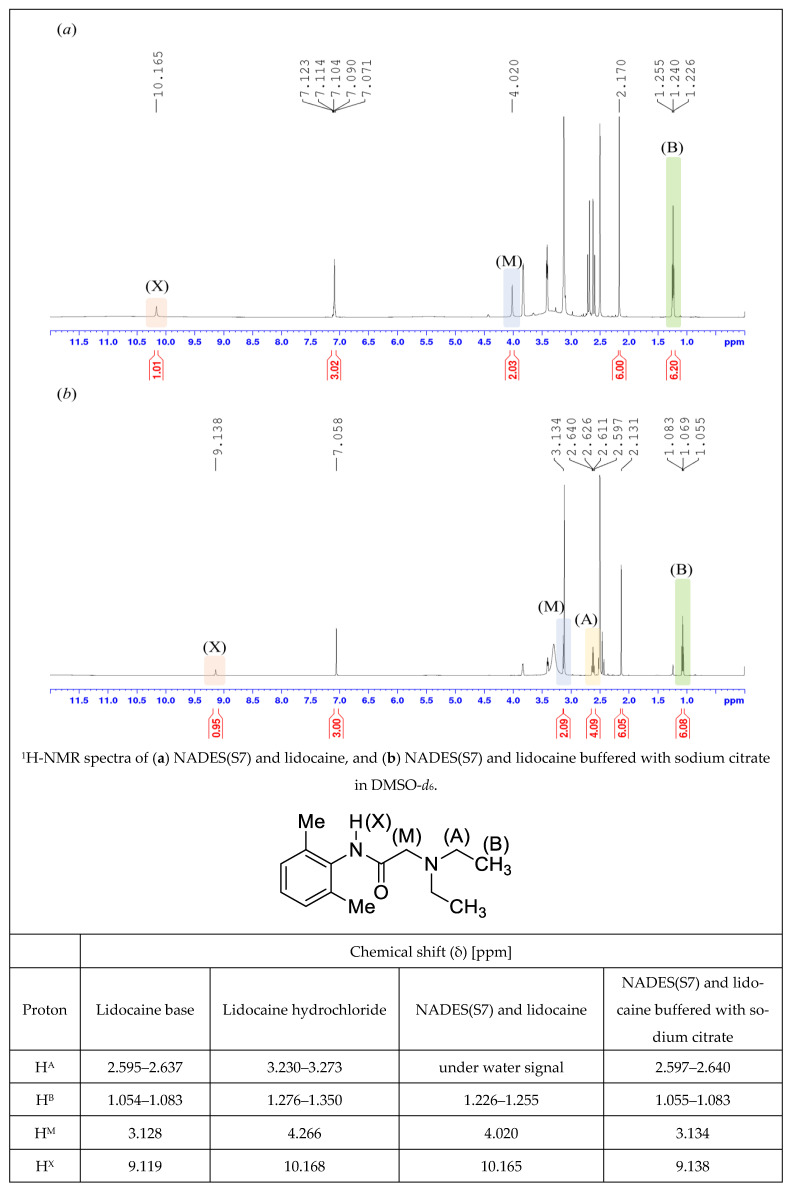
^1^H-NMR spectra and chemical shifts of the lidocaine-containing samples (**a**) NADES(S7) and lidocaine, and (**b**) NADES(S7) and lidocaine buffered with sodium citrate compared to both the lidocaine base and hydrochloride.

^1^H NMR studies were conducted to assess the chemical stability of lidocaine in NADES(S7). It is already established in the literature that lidocaine is susceptible to amide hydrolysis under certain conditions, yielding 2,6-dimethylaniline [59]. Based on these findings, two samples, lidocaine dissolved in NADES(S7) (Figure 7a and lidocaine dissolved in NADES(S7) and buffered with sodium citrate (Figure 7b), were measured by NMR, along with the raw materials lidocaine (Figure 6a) and NADES(S7) (Figure 6c) and the possible degradation product 2,6-dimethylaniline (Figure 6d). Due to the downfield chemical shifts of the protonated form of lidocaine, its hydrochloride salt was also measured by NMR (Figure 6b).

Degradation was not observable in either the unbuffered (Figure 7a) or buffered (Figure 7b) samples, as no unidentifiable signals or peaks of 2,6-dimethylaniline (an expected degradation product) appeared in the spectra. The proton signals of the N,N-diethylamino acetamide fragment of the active pharmaceutical ingredient showed downfield shifts in the spectra, which can be explained by the acidic character and/or solvation properties of the NADES-containing matrices, as it is listed and compared with both lidocaine base and hydrochloride spectra in Figure 7. Additionally, we have supported these data with the corresponding ^13^C NMR spectra to confirm and validate the results of the ^1^H spectra. The ^13^C spectra of the mixtures showed no sign of degradation or significant shifts, unlike the ^1^H spectra. The Supplementary Material (Figure S2a–i) contains figures related to the ^13^C NMR spectra and ^1^H spectra with the corresponding functional groups.

As was formerly observed in the Raman spectra, the peaks of the N,N-diethylamino acetamide moiety were altered during the NMR studies. In conclusion, these findings support the assumption that protonation and/or solvation mostly affected the molecule at its acetamide site, thus altering the spectroscopic properties of lidocaine. The highlighted protons in Figure 7 show different shifts, which can be attributed to the matrix effect of the NADES.

### 3.3. Results of In Vitro Release and In Vitro Permeation Tests

**Figure 8:**
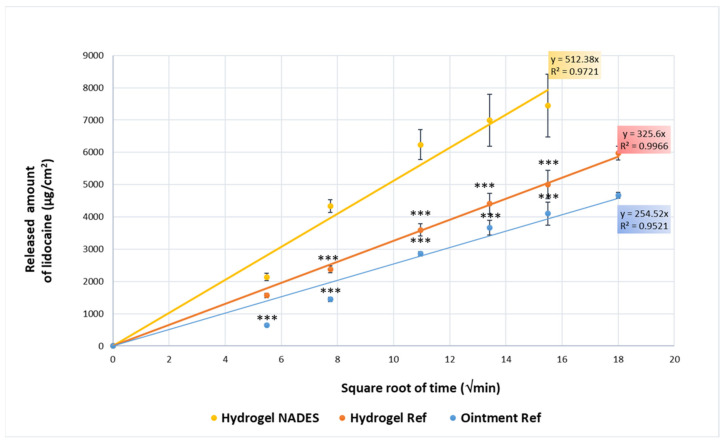
In vitro release profile of the cumulative amount of Hydrogel NADES compared with Hydrogel Ref and Ointment Ref (*** *p* < 0.001 vs. Hydrogel NADES).

The in vitro release test (IVRT) was conducted to investigate the release rate and extent of the lidocaine in the developed Hydrogel NADES compared to two reference formulations (Hydrogel Ref and Ointment Ref), following the EMA guideline on the quality and equivalence of locally acting cutaneous products [44]. The cumulative release of lidocaine followed the Higuchi diffusion model, showing a linear relationship with the square root of time [61,62], as depicted in Figure 8.

The Hydrogel NADES demonstrated the highest cumulative released amount of lidocaine with 7000 ± 1000 µg/cm^2^ after 6 h (slope = 512.38 µg/cm^2^ per √min, R^2^ = 0.9721), indicating the significant enhancement of lidocaine release compared with the Hydrogel Ref and Ointment Ref with 5900 ± 200 µg/cm^2^ (slope = 325.6 µg/cm^2^ per √min, R^2^ = 0.9966) and 4670 ± 90 µg/cm^2^ (slope = 254.52 µg/cm^2^ per √min, R^2^ = 0.9521) total released amount, respectively (Table 4). This result may be due to the pH-stabilizing effect of sodium citrate, which acts as a buffer and enhances the solubility of lidocaine in an acidic environment. Moreover, both reference formulations displayed a lower release profile, suggesting a more gradual drug release due to the absence of the pH-stabilizing agent and the higher pH value of the Hydrogel Ref (pH = 6.06), as well as the higher viscosity of the Ointment Ref (24,467 mPa·s, as previously published [63]).

These results indicate that the Hydrogel NADES formulation was significantly different from the Hydrogel Ref and Ointment Ref in terms of the amount of drug released, with the difference being highly statistically significant (*p* < 0.001). Moreover, the Hydrogel NADES provided the most rapid release of lidocaine, making it suitable for applications requiring fast therapeutic action. The cumulative release curves of the lidocaine formulations are reported in the Supplementary Material (Table S1).

**Figure 9:**
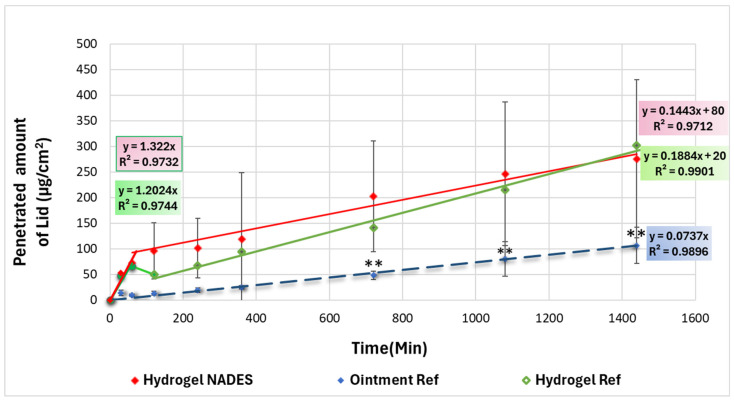
In vitro permeation test of the cumulative plot of the Hydrogel NADES compared with the Ointment Ref and Hydrogel Ref formulations (** *p* < 0.01 vs. Hydrogel NADES).

The in vitro permeation test (IVPT) results revealed similar lidocaine permeation profiles for each formulation over time (Figure 9).

All the formulations exhibited a two-step permeation pattern, with a rapid phase followed by a slow phase. The IVPT test suggested a non-Fickian permeation with different release rates due to factors such as the gelling agent interactions, varying ionized to unionized lidocaine ratios within the formulation, and the viscosity of the systems. This observation reflects a combination of an initially rapid and then a sustained effect. The IVPT parameters of the different formulations are summarized in Table 5.

The cumulative permeated amounts of the different formulations were evaluated after 24 h. The Hydrogel NADES (300 ± 100 µg/cm^2^) and Hydrogel Ref (300 ± 100 µg/cm^2^) showed similar results; however, the Ointment Ref (100 ± 40 µg/cm^2^) exhibited significantly lower permeation values. The Hydrogel NADES and Hydrogel Ref demonstrated a two-step permeation pattern, with a relatively fast initial permeation rate of 1.322 µg/cm^2^/min (R^2^ = 0.9732) and 1.2024 µg/cm^2^/min (R^2^ = 0.9744) at 60 and 30 min, respectively.

In the second phase, the permeation rate decreased, and the Hydrogel NADES exhibited a permeation rate of 0.1443 µg/cm^2^/min (R^2^ = 0.9712), which was similar to the permeation rate of the Hydrogel Ref of 0.1884 µg/cm^2^/min (R^2^ = 0.9901).

In contrast, the Ointment Ref showed a very low initial rapid permeation compared to the hydrogels, and the second sustained permeation phase was specific, with excellent linearity, as indicated by the high correlation coefficient (R^2^ = 0.9896), suggesting a consistent and predictable permeation process. In this case, just Permeation Rate 2 was calculated (0.0737 µg/cm^2^/min) due to the higher viscosity (24,467 mP·s, as previously published [63]) of the preparation.

Overall, hydrogels with the specific two-step profile of the skin permeation pattern of lidocaine can be described by the presence of two forms of lidocaine (uncharged and positively charged, protonated) in the aqueous formulations depending on the pH. According to the literature, the uncharged form, with higher hydrophobic properties, is able to penetrate membranes faster [64]. The initial fast permeation rate assumes more uncharged forms of lidocaine, but during the diffusion process of lidocaine from the donor phase to the acceptor phase, the pH of the donor phase changes to acidic, where the protonated form is more characteristic, which already shows slower skin permeation. In our study, the NADES seemed to promote the initial fast permeation of lidocaine.

### 3.4. The Effect of the Formulations on the Physiological Parameters of the Skin In Vivo

**Figure 10:**
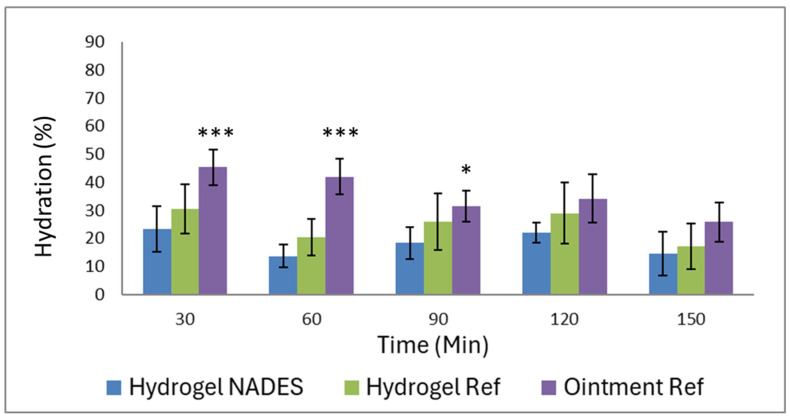
Hydration effect of the preparations (* *p* < 0.05, *** *p* < 0.001 vs. Hydrogel NADES at each measurement time).

Untreated skin can be classified into three groups within the normal range using the Corneometer^®^ [45,46]. According to this classification, skin hydration values below 30 corneometer units (c.u.) indicate very dry skin, values between 30 and 40 c.u. correspond to dry skin, and values above 40 c.u. indicate normal skin hydration. In our study, the Corneometer results showed that the initial skin hydration value of all the participants was between 31 and 56 c.u., so the skin condition can be classified as dry to normal. Figure 10 illustrates the moisturizing effect of the different formulations, with the moisture of untreated skin set to zero as a baseline. The subsequent changes in hydration were then expressed as percentages relative to this baseline. After application, the Ointment Ref achieved the highest peak hydration of 45.34 ± 15.44% at 30 min, which gradually decreased to 25.89 ± 17.06% at 150 min (compared to the baseline untreated skin moisture content), demonstrating superior moisturizing ability by achieving significantly higher hydration levels shortly after application. However, this intense moisturizing property was almost halved by the last measurement, resulting in a greater drop in the moisture levels over time. The Hydrogel Ref showed a moderate peak hydration of 30.43 ± 19.45% at 30 min, which decreased steadily to 17.19 ± 18.43% at 150 min. In contrast, the Hydrogel NADES exhibited the lowest peak hydration of 23.38 ± 18.32% at 30 min, with a gradual decrease to 14.50 ± 17.40% at 150 min. Its moisturizing effect was less pronounced than that of the reference formulations. Despite this result, the trend still demonstrated improved hydration for the hydrogel NADES formulation over time, suggesting its role as a skin-compatible option.

**Figure 11:**
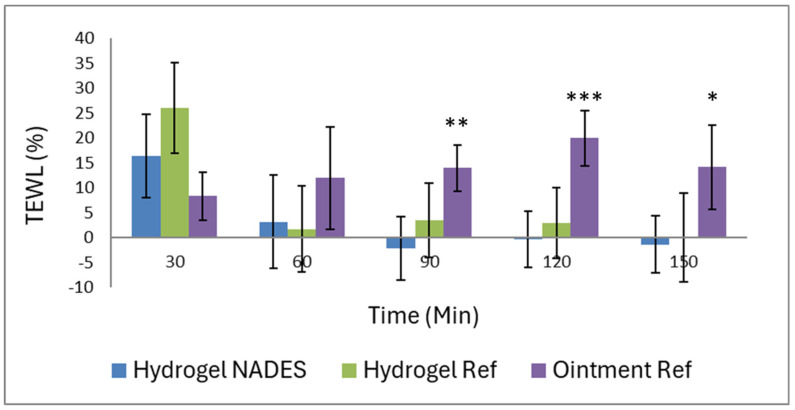
Effect of the preparations on the transepidermal water loss (TEWL) (* *p* < 0.05, ** *p* < 0.01, *** *p* < 0.001 vs. Hydrogel NADES at each measurement time).

Evaluation of the transepidermal water loss (TEWL) is a critical measurement for assessing the skin barrier function, as it provides insight into the skin’s ability to retain moisture and protect against external irritants. The Tewameter, similar to the Corneometer, establishes a baseline TEWL measurement of untreated skin and allows the assessment of the relative percentage reductions in the TEWL to determine the effectiveness of various formulations in maintaining the skin’s barrier function. In this study, the Hydrogel NADES demonstrated stable TEWL percentages over time, indicating minimal water loss and reliable skin barrier protection. As in Figure 11, the Hydrogel NADES showed an initial slight increase in TEWL at 30 min (16.28 ± 18.71%), but it stabilized near or below baseline until the end of 150 min (−1.04 ± 12.89%), indicating reliable skin barrier protection and consistent performance. The Hydrogel Ref displayed moderate variability, with the TEWL reaching a peak at 30 min (26.03 ± 20.33%) and stabilizing close to zero at later intervals. The Ointment Ref exhibited the highest TEWL percentages, particularly after 60 min, showing weaker barrier support and more significant water loss compared with the other two formulations.

Overall, both measurements indicate that the Ointment Ref exhibited superior hydration at the first two time points (both 30 and 60 min). This result may be due to the hygroscopic property of macrogol, which initially draws water into the skin. However, this effect was transient, and over time, the moisture-retaining ability of the ointment diminished. After 90 min, treatment with the ointment resulted in higher transepidermal water loss, which confirmed the irritative effect of macrogol. In contrast, the hydrogel formulations, particularly the Hydrogel NADES, provided more stable hydration that was maintained better over time and stabilized the TEWL near or below zero. These effects may be attributed to the matrix structure of the hydrogels, which allows for continuous hydration of the skin and provides a film layer, which helps retain the barrier function of the skin after prolonged application. Therefore, hydrogels are more suitable for ensuring long-term skin compatibility and barrier protection due to their sustained hydration effect. Further parameters related to hydration and TEWL are provided in the Supplementary Materials (Tables S2 and S3).

## 4. Conclusions

The primary disadvantages of local anesthetic injections are their invasive nature and the potential for pain during administration, which can increase the risk of systemic toxicity after the initial rapid onset and short duration. Despite the progress in the research and development of non-invasive dermal anesthetic formulations (creams, gels, ointments), they cannot meet the need to replace subcutaneous local anesthetic injections due to the long-term onset of action (30–60 min under occlusive dressing). This study successfully demonstrates the formulation of lidocaine-loaded NADES-containing hydrogels and highlights their potential for enhanced dermal delivery. The design of experiments (DoE) approach revealed that the concentrations of NADES and the pH-adjusting sodium citrate significantly influenced both the pH and the viscosity of the hydrogels. The high R-squared values for both the pH and viscosity models confirmed the strong and reliable relationship between the independent variables and the desired hydrogel properties, ensuring precision in formulation. Physicochemical investigations proved the chemical stability and compatibility of the lidocaine in the NADES and NADES-containing hydrogel systems. In vitro release and permeation studies showed a higher release rate and better permeation through the epidermis. Additionally, the in vivo physiological effect of the developed hydrogel illustrated a favorable effect on the skin barrier compared with traditional formulations. Hydrogels containing NADES formulations have the potential to address these challenges in minor surgeries, such as aesthetic procedures, local anesthesia, and chronic pain management. The rapid initial drug permeation may provide a shorter onset of action, followed by a slower penetration to maintain the therapeutic effect. In addition, the hydrogel formulation maintained the moisturizing effect without irritating the skin. Conducting animal studies could facilitate a more detailed investigation of these findings.

## Figures and Tables

**Table 1:** Detailed summary of various eutectic solvents capable of dissolving lidocaine.

Sample	Compound 1	Compound 2	Compound 3	Molar Ratio	Added Water Content (*w*/*w*%)
S1	Citric acid	Glucose	-	1:1	20%
S2	Citric acid	Saccharose	-	1:1	20%
S3	Lactic acid	Glucose	Water	5:1:3	-
S4	Lactic acid	Fructose	-	5:1	-
S5	Choline chloride	Lactic acid	-	1:4	-
S6	Malic acid	Betaine	Water	2:1:5	-
S7	Choline chloride	Citric acid	-	1:1	20%

**Table 2:** Experimental design, values, and levels of independent variables.

Determinants	Code	Lower Level (−1)	Mid-Level (0)	Upper Level (+1)
Independent variables				
NADES *w*/*w*%	(X_1_)	10	15	20
Sodium citrate *w*/*w*%	(X_2_)	8	13	18
Dependent variables				
pH	(Y_1_)			
Viscosity (Pa·s)	(Y_2_)			

**Table 3:** Independent and dependent variables of the DoE samples.

Samples	(X_1_) NADES (s7) (*w*/*w*%)	(X_2_) Na Citrate (*w*/*w*%)	(Y_1_) pH	(Y_2_) Viscosity (mPa·s)
N1	10 (−1)	8 (−1)	4.46 ± 0.02	11,000 ± 1000
N2	10 (−1)	13 (0)	4.84 ± 0.05	14,000 ± 1000
N3	10 (−1)	18 (+1)	5.10 ± 0.10	340 ± 30
N4	15 (0)	8 (−1)	3.92 ± 0.02	11,200 ± 400
N5	15 (0)	13 (0)	4.4 ± 0.11	16,200 ± 900
N6	15 (0)	18 (+1)	4.67 ± 0.02	600 ± 200
N7	20 (+1)	8 (−1)	3.63 ± 0.00	13,700 ± 700
N8	20 (+1)	13 (0)	4 ± 0.06	18,000 ± 1000
N9	20 (+1)	18 (+1)	4.34 ± 0.02	1700 ± 400

**Table 4:** Release parameters of Hydrogel NADES compared with Hydrogel Ref and Ointment Ref.

Sample	Time (min)	Cumulative Drug Release (µg/cm²) ± SD	R^2^	Release Constant µg/cm² per √min
Hydrogel NADES	360	7000 ± 1000	0.9721	512.38
Hydrogel Ref	360	5900 ± 200	0.9966	325.6
Ointment Ref	360	4670 ± 90	0.9521	254.52

**Table 5:** Permeation parameters of the Hydrogel NADES compared with the Ointment Ref and Hydrogel Ref formulations.

Sample	Time (min)	Cumulative Drug Permeation (µg/cm^2^) ± SD	R^2^ 1	R^2^ 2	Permeation Rate 1 (µg/cm^2^/min)	Permeation Rate 2 (µg/cm^2^/min)
Hydrogel NADES	1440	300 ± 100	0.9732	0.9712	1.322	0.1443
Hydrogel Ref	1440	300 ± 100	0.9744	0.9901	1.2024	0.1884
Ointment Ref	1440	110 ± 40	0.9896	-	-	0.0737

## Data Availability

Data is contained within the article or Supplementary Materials.

## Supplementary Materials

The following supporting information can be downloaded at https://www.mdpi.com/article/10.3390/pharmaceutics17030324/s1, Figure S1: Viscosity curves of hydrogel formulations from the Design of Experiments (DoE) with varying concentrations of NADES and sodium citrate; Figure S2: ^13^C NMR spectra and ^1^H NMR spectra with corresponding functional groups; Table S1: Results of cumulative amount of lidocaine in each sampling time (IVRT); Table S2: Hydration values in all evaluating times (mean ± SD); Table S3: TEWL values in all evaluating times (mean ± SD).
