# Supplementary material for: The Role of Natural Deep Eutectic Solvents in a Hydrogel Formulation Containing Lidocaine
Source: Pharmaceutics. 2025 Mar 2;17(3):324. doi: 10.3390/pharmaceutics17030324 (PMC11944536; doi:10.3390/pharmaceutics17030324)
Supplement: Supplementary file 1 [file pharmaceutics-17-00324-s001.zip › pharmaceutics-3469037-supplementary.pdf]

pharmaceutics

Supplementary Materials: The Role of Natural Deep Eutectic Solvents in a Hydrogel Formulation Containing Lidocaine

Feria Hasanpour, Mária Budai-Szűcs, Anita Kovács, Rita Ambrus, Orsolya Jójárt-Laczkovich, Boglárka Szalai, Branimir Pavlić, Péter Simon, Levente Törteli and Szilvia Berkó

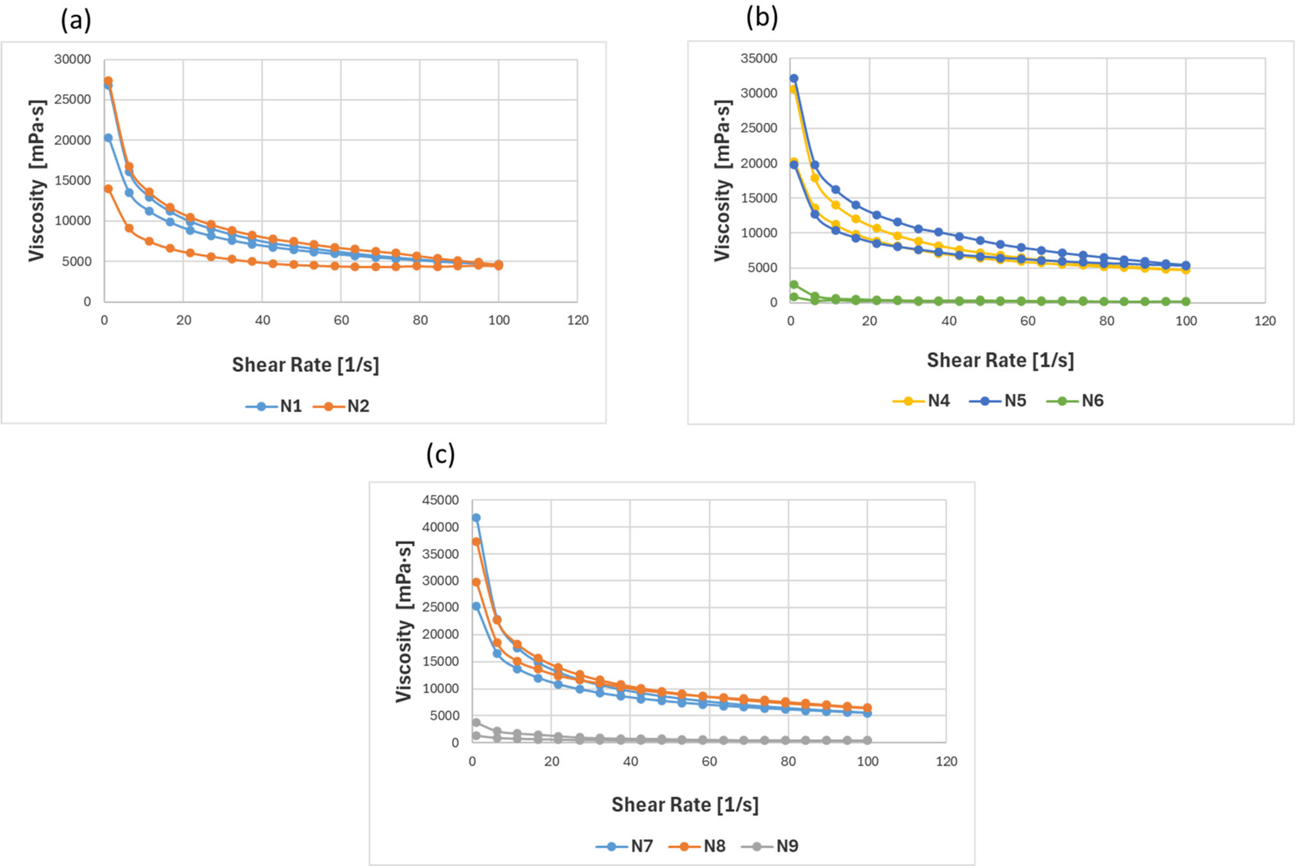

Figure S1. Viscosity curves of hydrogel formulations from the Design of Experiments (DoE) with varying concentrations of NADES and sodium citrate .

https://doi.org/10.3390/pharmaceutics17030324

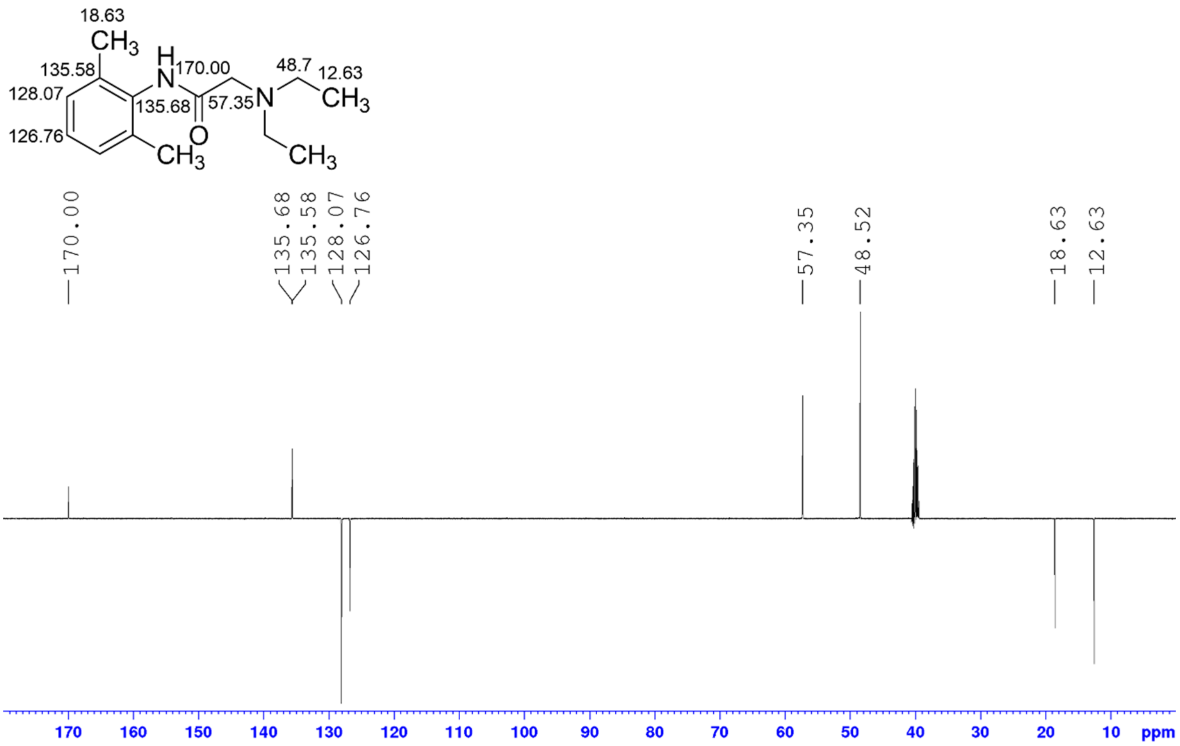

Figure S2a. ^{13}C-NMR spectrum of lidocaine base in DMSO-*d*6.

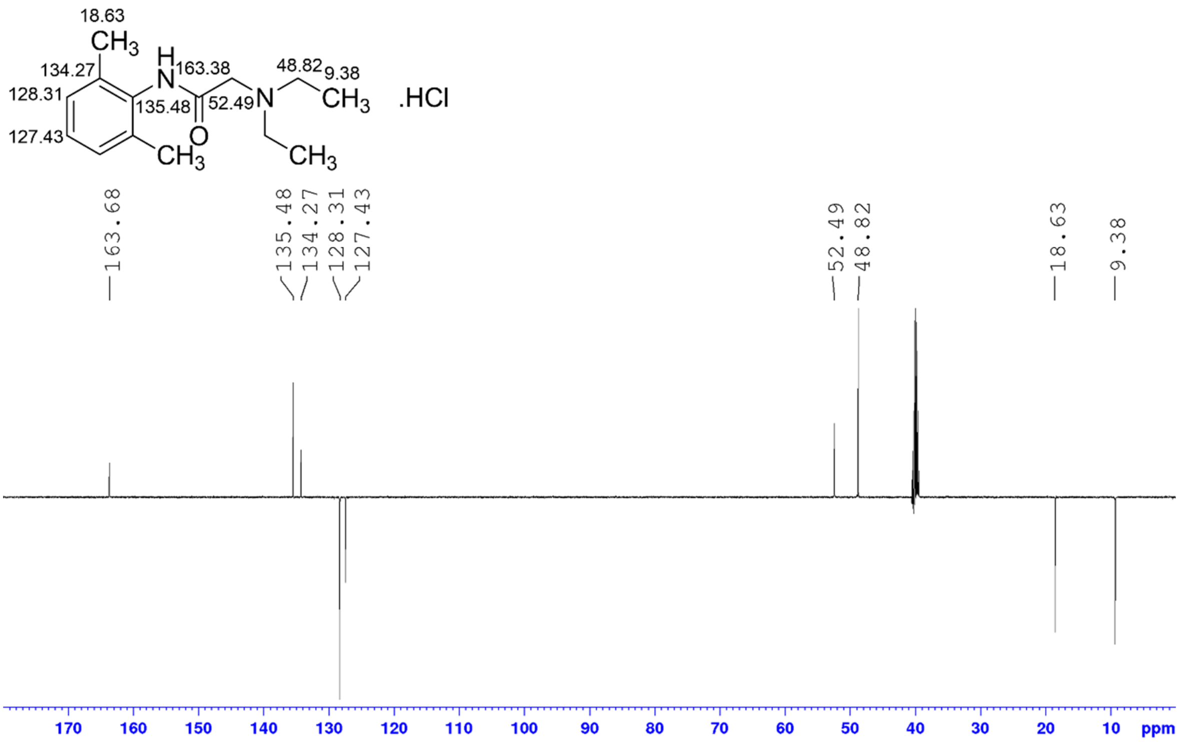

Figure S2b. ^{13}C-NMR spectrum of lidocain hydrochloride in DMSO-*d*6.

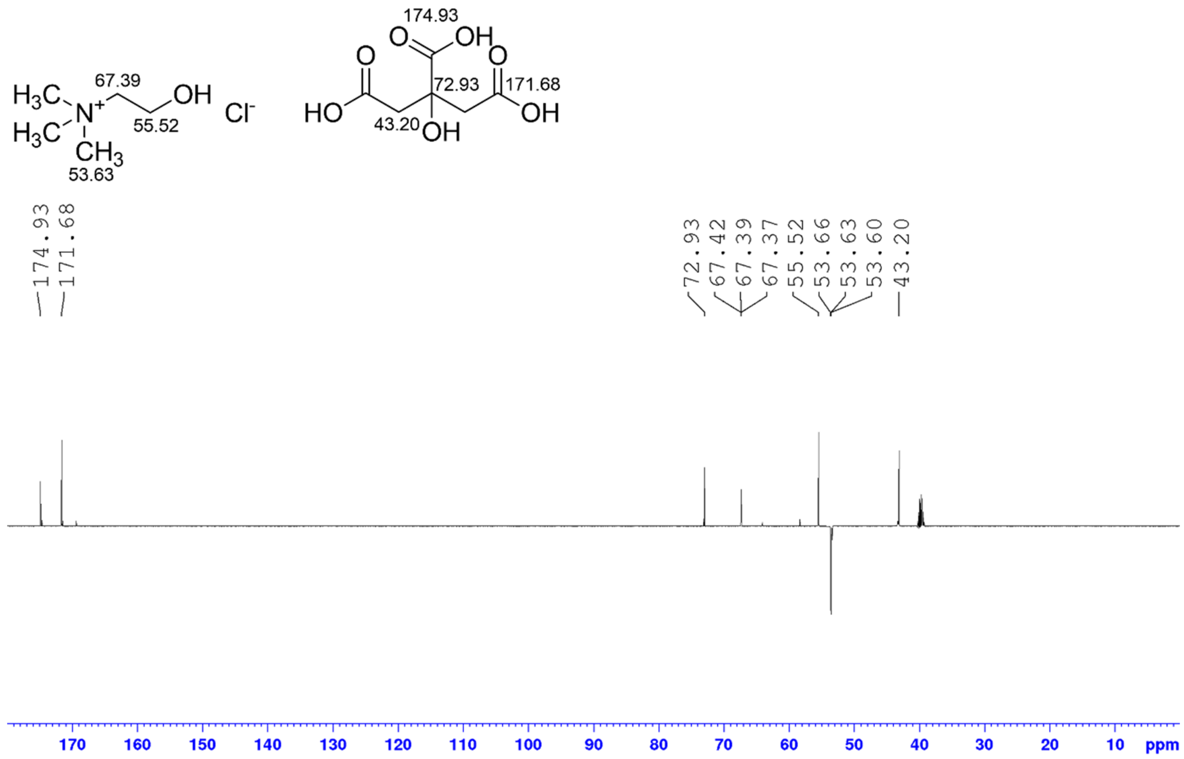

Figure S2c. ^{13}C-NMR spectrum of NADES (S7) in DMSO-*d6*.

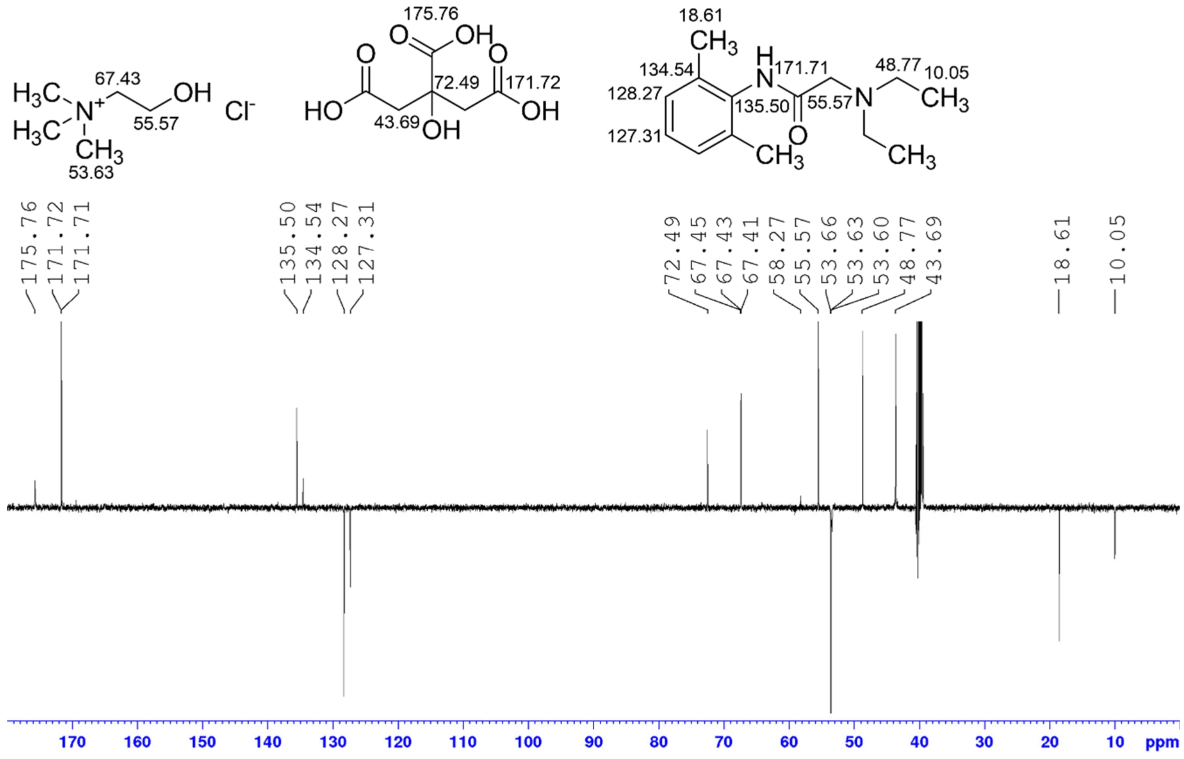

Figure S2d. ^{13}C-NMR spectrum of NADES (S7) and lidocain base in DMSO-*d6*.

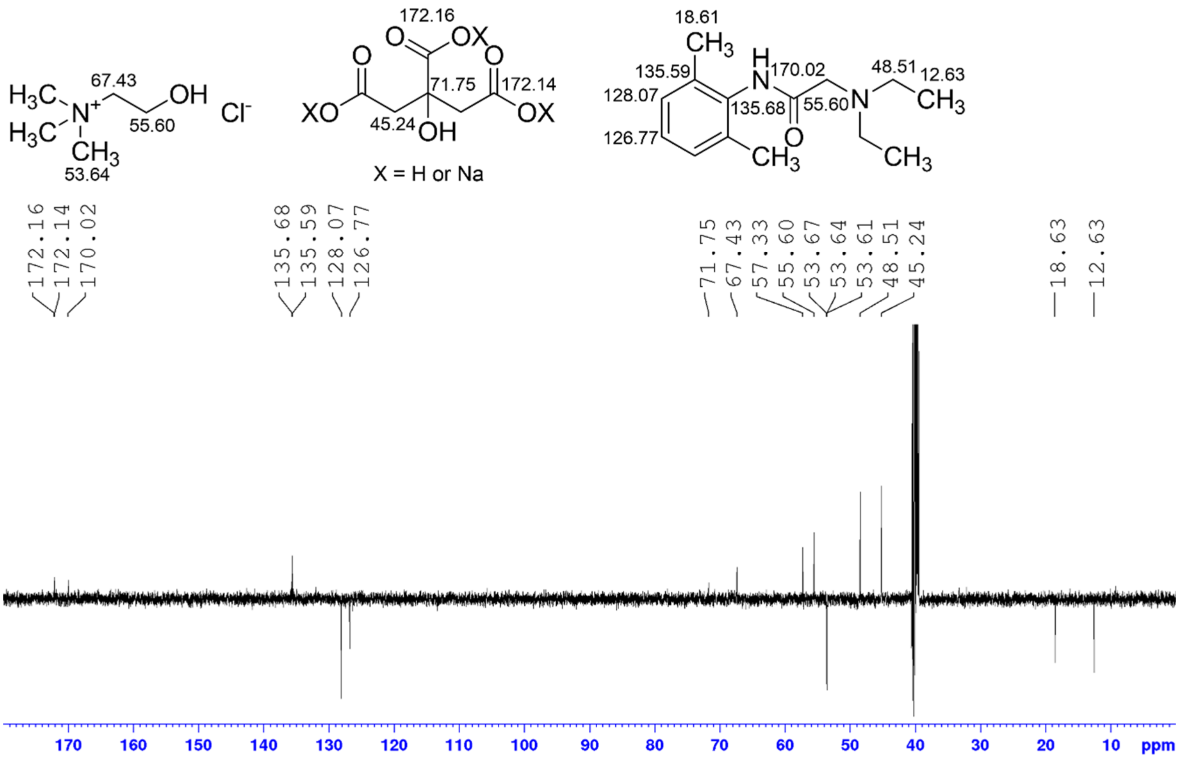

Figure S2e. ^{13}C-NMR spectrum of NADES(S7) and lidocaine buffered with sodium citrate in DMSO-*d*6.

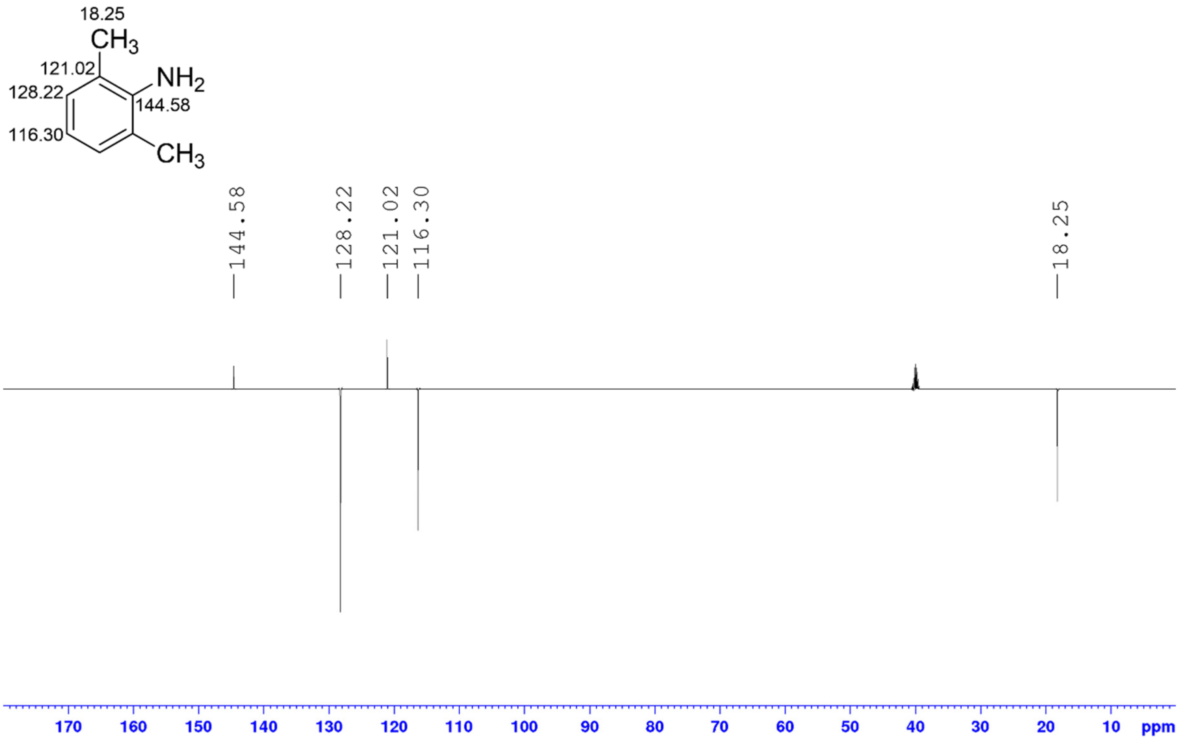

Figure S2f. ^{13}C-NMR spectrum of 2,6-dimethylaniline in DMSO-*d*6.

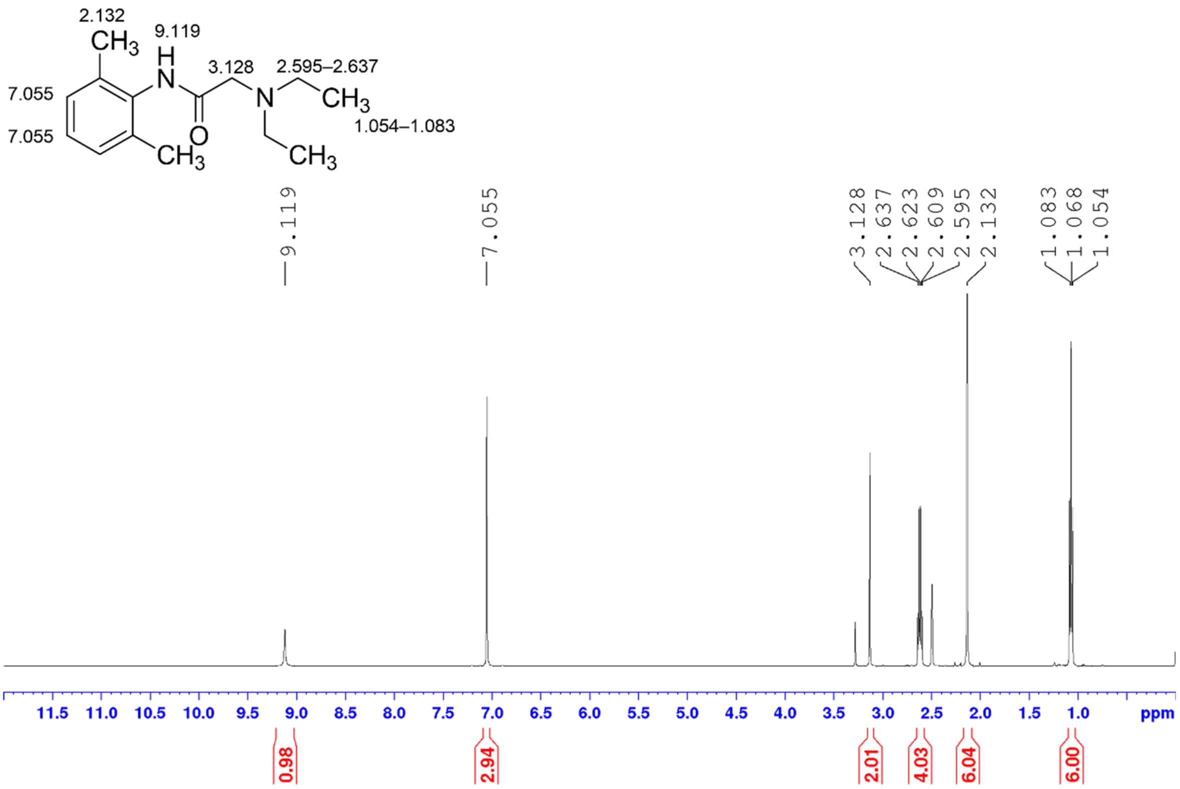

Figure S2g. ^{1}H-NMR spectrum of lidocaine base in DMSO-d_6.

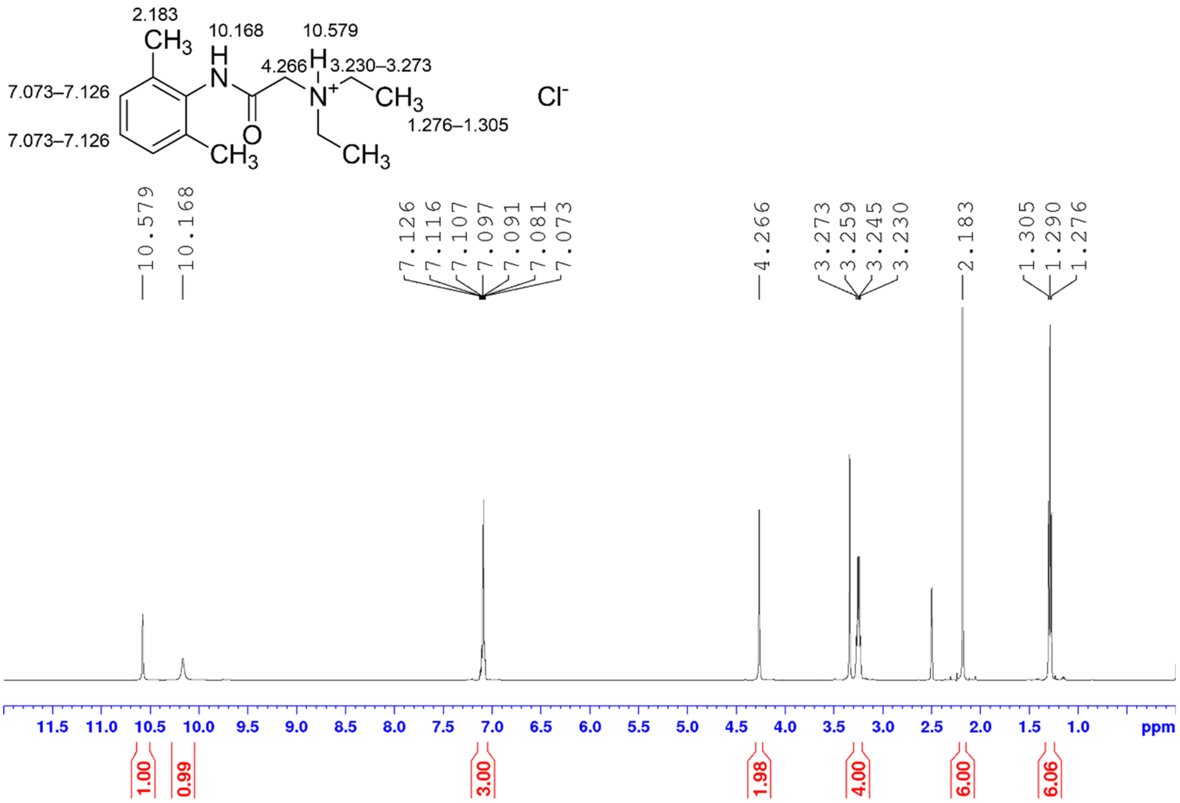

Figure S2h. ^{1}H-NMR spectrum of lidocain hydrochloride in DMSO-d_6.

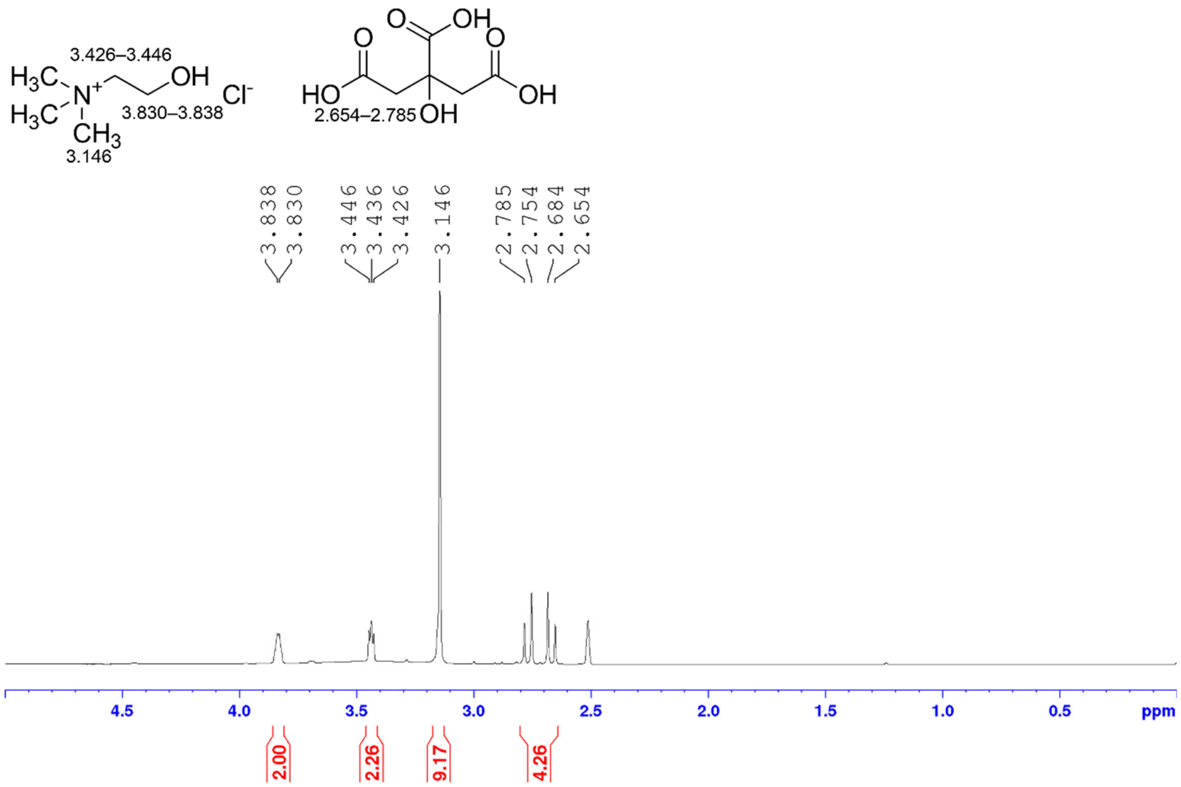

Figure S2i. ^{1}H-NMR spectrum of NADES (S7) in DMSO-d_6.

Table S1. Results of cumulative amount of lidocaine in each sampling time (IVRT).

		Hydrogel NADES		Hydrogel Ref		Ointment Ref	
Time(min)	square root of time (min)	Average (µg/cm²)	SD	Average (µg/cm²)	SD	Average (µg/cm²)	SD
30	5.48	2137.08	108.91	1564.35	71.96	649.60	36.77
60	7.75	4328.16	196.98	2373.78	109.94	1441.19	66.92
120	10.95	6238.04	465.60	3595.33	192.74	2856.35	65.81
180	13.42	6992.76	801.79	4412.58	321.95	3663.84	230.88
240	15.49	7449.82	969.72	5003.58	436.87	4105.61	357.22
360	18.97	7319.40	1190.20	5946.01	211.32	4668.14	88.41

Table S2. Hydration values in all evaluating times (mean ± SD).

	Hydrogel Ref		Hydrogel NADES		Ointment Ref	
**Time(min)**	Mean hydration (%)	SD	Mean hydration (%)	SD	Mean hydration (%)	SD
**30**	30.43	19.45	23.38	18.32	45.34	15.44
**60**	20.44	14.47	13.77	9.28	42.01	15.23
**90**	26.01	22.47	18.35	12.72	31.52	13.36
**120**	28.94	24.44	22.06	8.28	34.21	21.19
**150**	17.19	18.43	14.50	17.40	25.89	17.06

Table S3. TEWL values in all evaluating times (mean ± SD).

	Hydrogel Ref		Hydrogel NADES		Ointment Ref	
**Time(min)**	Mean TEWL (%)	SD	Mean TEWL (%)	SD	Mean TEWL	SD
**30**	26.03	20.33	16.28	18.710	8.30	10.73
**60**	1.69	19.26	3.16	21.02	11.95	22.95
**90**	3.44	16.51	-2.24	14.25	13.97	10.31
**120**	2.89	15.98	-0.34	12.72	19.94	12.49
**150**	0.02	19.95	-1.04	12.89	14.11	18.94
